# Operational Load Monitoring of a Composite Panel Using Artificial Neural Networks

**DOI:** 10.3390/s20092534

**Published:** 2020-04-29

**Authors:** Waldemar Mucha, Wacław Kuś, Júlio C. Viana, João Pedro Nunes

**Affiliations:** 1 Department of Computational Mechanics and Engineering, Silesian University of Technology, 44-100 Gliwice, Poland; waclaw.kus@polsl.pl; 2 Department of Polymer Engineering, IPC—Institute for Polymers and Composites, University of Minho, 4800-058 Guimarães, Portugal; jcv@dep.uminho.pt (J.C.V.); jpn@dep.uminho.pt (J.P.N.)

**Keywords:** operational load monitoring, artificial neural networks, structural health monitoring, finite element method, strain measurement, omega-stiffened composite panel

## Abstract

Operational Load Monitoring consists of the real-time reading and recording of the number and level of strains and stresses during load cycles withstood by a structure in its normal operating environment, in order to make more reliable predictions about its remaining lifetime in service. This is particularly important in aeronautical and aerospace industries, where it is very relevant to extend the components useful life without compromising flight safety. Sensors, like strain gauges, should be mounted on points of the structure where highest strains or stresses are expected. However, if the structure in its normal operating environment is subjected to variable exciting forces acting in different points over time, the number of places where data will have be acquired largely increases. The main idea presented in this paper is that instead of mounting a high number of sensors, an artificial neural network can be trained on the base of finite element simulations in order to estimate the state of the structure in its most stressed points based on data acquired just by a few sensors. The model should also be validated using experimental data to confirm proper predictions of the artificial neural network. An example with an omega-stiffened composite structural panel (a typical part used in aerospace applications) is provided. Artificial neural network was trained using a high-accuracy finite element model of the structure to process data from six strain gauges and return information about the state of the panel during different load cases. The trained neural network was tested in an experimental stand and the measurements confirmed the usefulness of presented approach.

## 1. Introduction

Operational load monitoring (OLM) is a process of measuring the number and characteristics of load cycles to which a structure is submitted in its normal operating service environment. The purpose is to predict the remaining lifetime of the structure in-service [1]. A related process to OLM is structural health monitoring (SHM) which consists of damage diagnosis and reduction of system performance prognosis. In SHM, data from a sensorized structure are gathered in order to find potential changes in geometry, material parameters (damage, properties), boundary conditions, and system connectivity [2,3,4,5]. Sometimes, OLM is also referred to as an element of SHM [6].

OLM is particularly useful in aeronautical and aerospace industries [7,8] where continual monitoring allows one to reduce the costs of maintenance actions (assessment of structural components condition based on visual inspections, X-ray analysis, eddy-current testing, etc.) and increase aircraft availability (aircraft does not have to be taken out of service for maintenance and it may have a prolonged life) [1]. Other benefits, besides reduction of costs and fewer aircraft on ground, are improved flight safety, mission reliability and effectiveness, improved aircraft performance, and reduced fuel consumption and pollution [9].

In OLM, the data is usually gathered from embedded or surface mounted strain sensors. Composite aerostructures allow to implement intrinsic optical fiber sensors in order to obtain strain distribution [10,11,12]. The data can be also obtained from strain gages. In damage detection and load monitoring of aerostructures, there is a limited number of strain sensors, mounted in critical points. For rough estimation of loads in other locations, on-line monitoring of flying parameters (velocity, altitude, mass, and acceleration) is performed [13]. Therefore, the accuracy of OLM is based on the number of strain sensors.

However, if the structure in its normal operating environment is subjected to variable exciting forces acting in different locations and directions over time, the number of critical points from where data should be acquired largely increases. This can cause a decrease in the accuracy of OLM system, if the number of critical points is too high to perform strain measurement from all of them [14].

The idea of the authors is to perform strain measurements in only a few critical points and estimate the state of the whole structure using artificial intelligence techniques. Artificial neural networks (ANNs) often work very well as fast numerical approximators [15,16,17,18,19,20]. With an accurate numerical model and knowledge of possible magnitude range and location of loads in operating environment of the structure, training data for ANN can be generated. The ANN could be trained to return maximal stresses, strains, force locations, or deformations of the structure based on strain data acquired from few sensors.

Composite structures are often used in the aerospace industry because of their efficiency (minimal weight and high stiffness) [21,22]. Typical composite lightweight aerostructures are sandwich laminates (e.g., with honeycomb, truss, or foam cores) [23] and stiffened composite panels (with stiffeners of several shapes, e.g., blades, T-bars, hats, and omega) [24,25]. In the following paper, an omega-stiffened carbon/epoxy woven composite panel is used to verify and validate the concept of utilizing ANNs in OLM. For strain measurement, strain gauges are used. Finite element (FE) model of the panel was built and its accuracy was assessed. The FE model was used to generate data to train the ANN. The details are described in Section 2. In Section 3, the effectiveness of the ANN is presented—values on the output of ANN, where experimental data is given as inputs, are compared to the theoretical values from FE model. Two cases are considered, where the ANN is tested off-line with the experimental data acquired in advance, and where the ANN is tested in real time at the experimental stand. The results are discussed in Section 4 and the work is concluded in Section 5.

## 2. Materials and Methods

**Figure 1:**
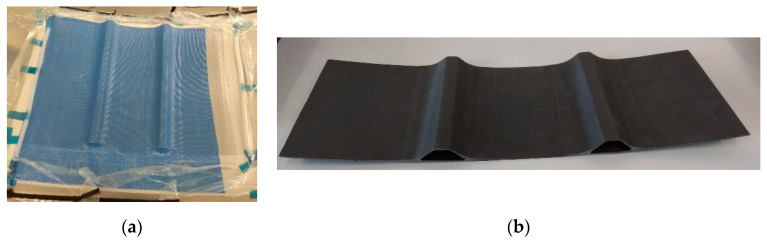
Considered composite panel: (**a**) manufacturing process and (**b**) final effect.

A prototype of typical structure applied in aerospace—a composite omega-stiffened panel made of carbon/epoxy woven laminate—was manufactured using vacuum infusion process [26,27]. The process, presented in Figure 1a, consisted of filling the voids in the stack of carbon woven fabric (laid on a mold and sealed with flexible non-permeable film) with liquid resin that is driven by vacuum pressure through special tubing. The overall dimensions of the manufactured panel (Figure 1b) are 597 mm × 204 mm × 29 mm (the curvature of the panel is of radius 1620 mm).

**Figure 2:**
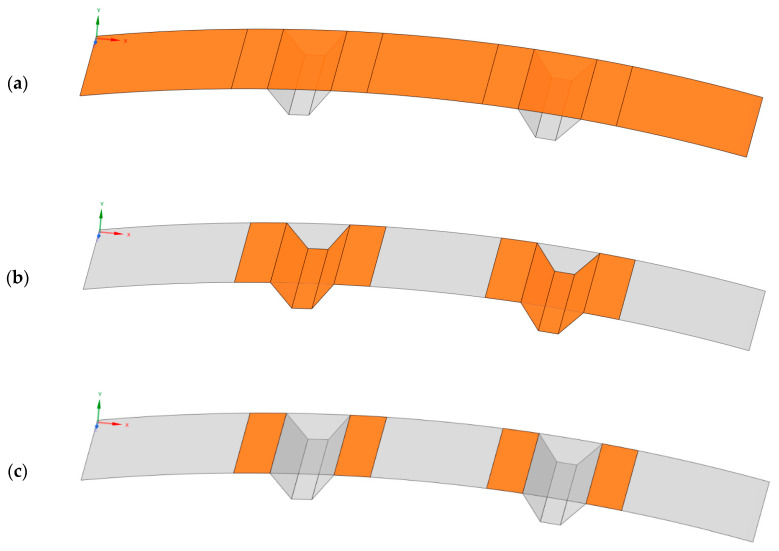
Geometry of the omega-stiffened composite panel: (**a**) base part; (**b**) reinforcement part; and (**c**) double-thickness part.

The geometry of the panel can be divided to two parts: base and reinforcement (Figure 2a,b). Each part consists of 10 layers of carbon woven with different ply orientation (presented in Table 1, even symmetry is applied). Each single ply is 0.23 mm thick and therefore, if the geometry was presented as surface model, most of the surfaces would be 2.3 mm thick, except four surfaces common to the base and reinforcement parts, highlighted in Figure 2c, that would be 4.6 mm thick.

The mechanical properties of a single orthotropic ply [28] and those ones calculated [29] for the laminate are presented in Table 2. An orthotropic material is described by the following constants: three Young’s modules *E_i_* along axis *i*, three shear modules G*_ij_* in direction *j* on the plane normal to *i*, and three Poisson’s ratios *ν_ij_* that describe the contraction in direction *j* when extension is applied in direction *i* [30], where i,j∈{x,y,z} (*x* and *y* are in-plane axes, and *z* is normal to the plane). The properties of reinforcement part are identical as of single ply as all the layers have the same orientation.

**Figure 3:**
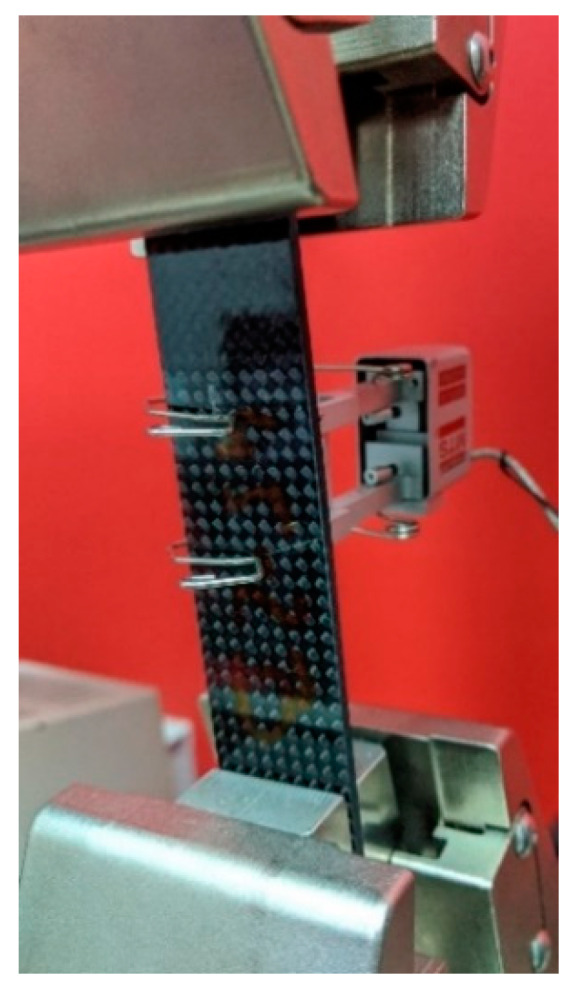
Photograph of experimental verification of computed mechanical properties of the composite rectangular samples.

Experimental verification of the calculated mechanical properties was performed. 10 rectangular samples of base material, 25 mm wide and 120 mm long (of thickness 2.3 mm), were prepared, where 5 of them were at orientation 0°/90° and 5 of them at orientation −45°/45°. The samples were subjected to static tension test at a crosshead speed of 0.5 mm/min and room temperature of 23 °C, according to the standard D 3039/D 3039M, in order to obtain Young Modulus Ex. The tests were performed using universal testing machine MTS Insight 10 equipped with a 10 kN load cell, by using a contact extensometer (Figure 3) to measure the sample displacement. The results of the tests are presented in Table 3.

**Figure 4:**
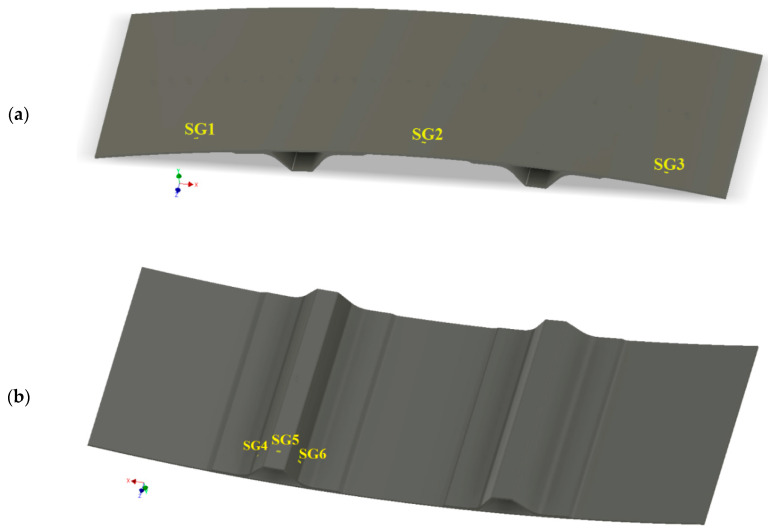
Mounting locations of strain gauges: (**a**) view from top and (**b**) view from bottom.

It is assumed that strain measurements are made in 6 points (in the longitudinal direction) of the panel (that will be performed in the experiment by means of strain gauges): SG1-SG6. Mounting areas of the strain gages are shown in Figure 4. The strain gauges are mounted in the very middle of each surface in the longitudinal direction, with the offset from the long edge of the panel of 19 mm.

**Figure 5:**
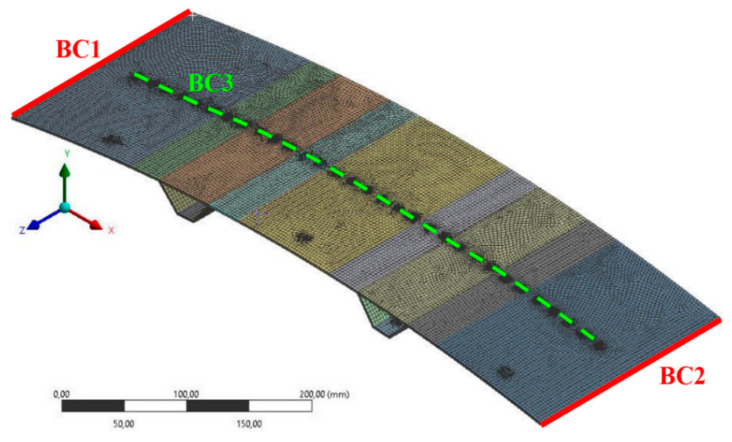
Mesh and boundary conditions of the finite element model of the omega-stiffened composite panel.

A finite element (FE) model of the considered panel was prepared, using ANSYS Workbench 2019 R3 software [31], with Composite PrepPost (ACP) module [32]. The mesh and boundary conditions are presented in Figure 5. The model consists of 26,357 surface elements of quadratic order, and 79,217 nodes. The boundary conditions, according to the experimental setup, are as follows (Figure 5): on the edge BC1 displacements along axes *X* and *Y* are fixed, on the edge BC2 displacements along axis *Y* are fixed, and it is assumed that the panel can be loaded along axis −*Y* at any point on the symmetry line BC3 where 31 round surfaces of diameter 5 mm, with distance between the centers of two adjacent surfaces of 17.5 mm, were isolated (according to the experimental loading bar)—displacements along axis *Z* are fixed on the same surface where load is applied in a given analysis. The global size of each finite element is 3 mm, refinement is applied at round surfaces on line BC3 and at separated surfaces corresponding to the strain gages positions.

**Figure 6:**
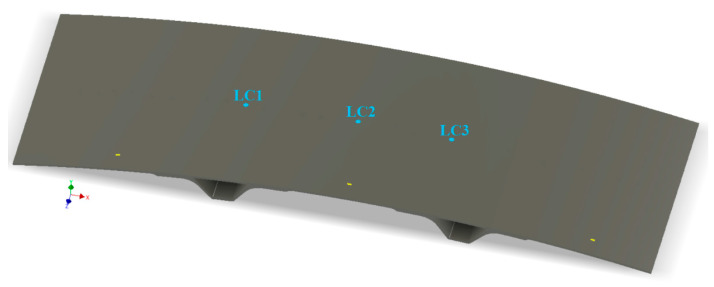
Load points in experimental verification.

**Figure 7:**
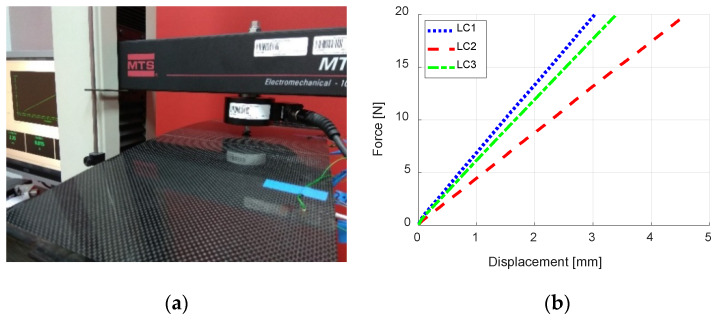
Experimental verification of the accuracy of numerical model: (**a**) photograph from the experiment and (**b**) force–displacement plots.

The accuracy of the FE model was verified experimentally. Three load cases were taken into consideration. Force was applied along axis −*Y* (in the range 0–20 N) sequentially in the middle of the rib (point LC1), in the middle of the composite panel (point LC2) and on the side of the rib (point LC3)—as presented in Figure 6. The experiment was performed using universal testing machine MTS Insight 10 with 500 N load cell (Figure 7a). In order to mimic the boundary conditions applied in the numerical model, a special steel frame of high stiffness, on which the composite panel was lying during the experiment, was manufactured and used. The test velocity was 0.5 mm/m, during the test data from general purpose strain gauges EA-13-125BT-120 [33], produced by Vishay Precision Group, were gathered (with 20 Hz sampling frequency) using data acquisition system Hottinger Baldwin Messtechnik (HBM) MGCplus [34] and Catman software. The testing machine MTS was connected with the data acquisition system MGCplus via analog output monitor/analog input card to record the force signal from the load cell.

**Figure 8:**
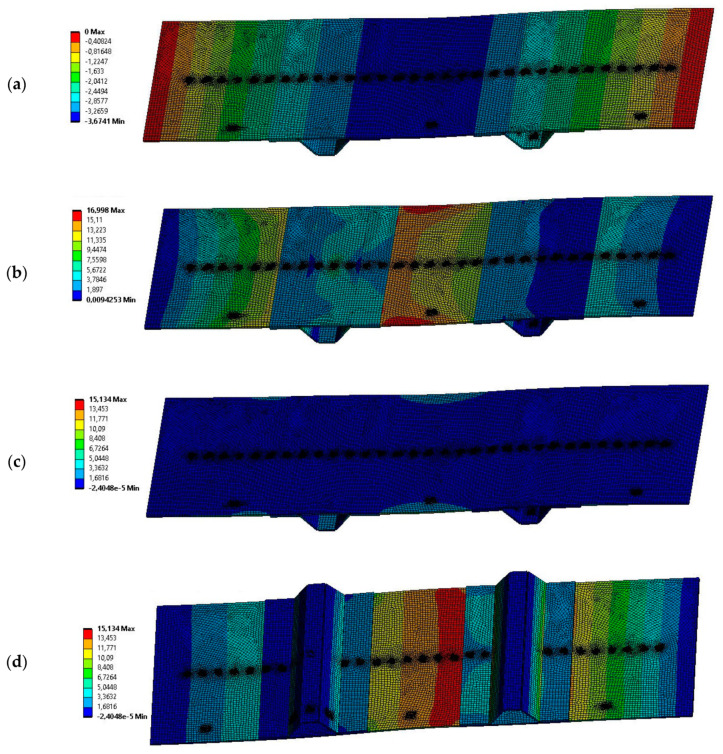
Example numerical results (20 N of force applied at point LC1): (**a**) directional deformation along axis *Y*; (**b**) von Mises stress; (**c**) maximum principal stress (view from top); and (**d**) maximum principal stress (view from bottom).

The first stage of experimental verification was to compare the bending stiffnesses obtained from numerical model *k*_num_ with the experimental ones *k*_exp_, by using the vertical displacements of points where load was applied. The force-displacement plots obtained from the experiment are presented in Figure 7b, being the displacement read from the machine traverse movement. Comparison of the bending stiffnesses obtained from numerical simulation and experiment (Spreadsheets S1–S3) are presented in the Table 4. In the FE simulations, the average vertical displacement of adequate round surface (on top of the model with the diameter of 5 mm) was noted for a single force value (the type analysis was linear-elastic). When calculating the stiffnesses from experimental data, linear approximations of the curves from Figure 7b were determined and their slopes were considered as the bending stiffnesses. Example results from FE analysis, where vertical force of magnitude 20 N was applied to point LC1, are presented in Figure 8.

**Figure 9:**
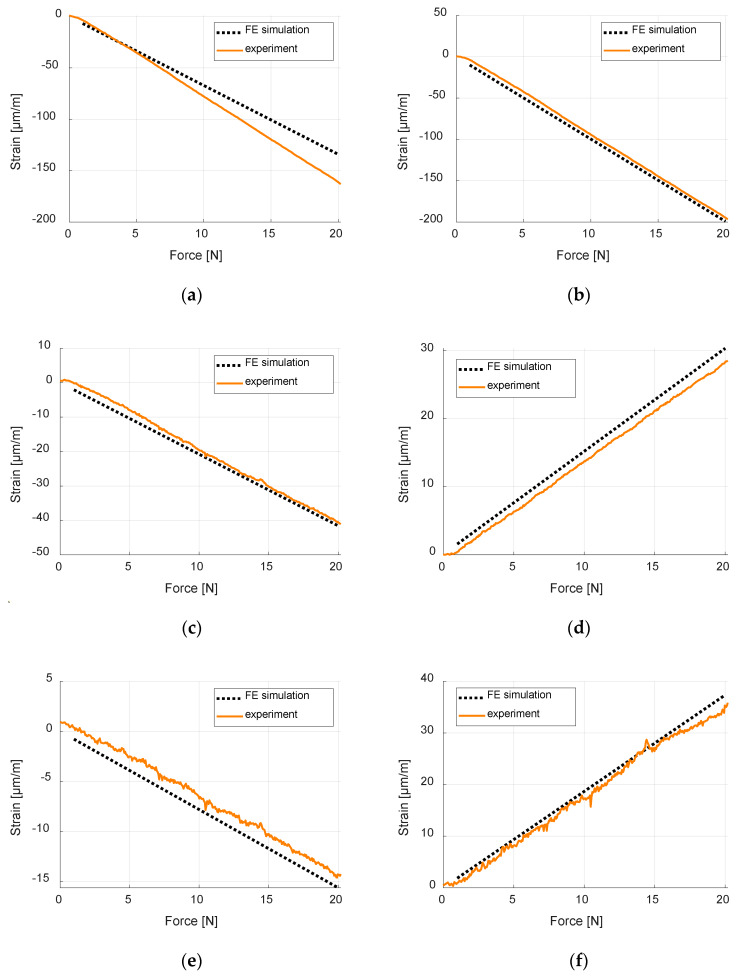
Comparison of numerical and experimental strain vales during load lase LC1: (**a**) strain gauge SG1; (**b**) strain gauge SG2; (**c**) strain gauge SG3; (**d**) strain gauge SG4; (**e**) strain gauge SG5; and (**f**) strain gauge SG6.

The second stage of experimental verification was to compare the strain values obtained from FE model (average values for surfaces of areas corresponding to size of strain gauges) with values measured by the mounted strain gauges during the experiment (Spreadsheets S4–S6). The root-mean-square errors for 20 strain values corresponding to integer loads from 1 to 20 N are presented in Table 5. Example strain results, for load case LC1, from numerical simulation and experiment, are presented in Figure 9.

This verification phase allowed to assess the quality of the FE model, which is representative of the experimental tests. The mechanical properties of the composite rectangular samples (0/90° and 45°/45°) are computed with a maximum error of the order of 5%. The stiffness of the composite panel is computed with an error less than 1.5%. Major deviations on computed strains occurred for SG1 with an error of 14.83 μm/m in load case however, average error of all strain gauges for all load cases was only 3.18 μm/m. Being verified, it has been concluded that the FE model was of sufficient accuracy to be used to train the ANN, without the necessity for model updating to refine the accuracy.

## 3. Results

Reference data, to train the ANN, were generated from numerical simulations of the panel. There were 1240 simulations performed where vertical loads of values 1, 2, 3, …, 40 N were applied to every of 31 round surfaces on line BC3 (see Figure 5), subsequently. For every simulation, the area average strain values corresponding to strain gauges SG1–SG6, and maximum inverse safety factor (ISF) values of the whole model were calculated and saved (Spreadsheet S7). If safety factor was defined as an indication of the margin to failure, i.e., if the applied load multiplied by it determines the failure load, a possible failure would occur when the safety factor was less than 1. In this case, the ISF would be the inverse number of the safety factor (possible failure to occur when the ISF is greater than 1). To assess the ISF, three safety criteria were taken into consideration—maximum stress, maximum strain, and Tsai–Wu criterion. The stress and strain limits for the first two criteria are listed in Table 6. These criteria are defined as the quotient of stresses and strains occurring in the structure and defined limits. The Tsai–Wu criterion (a widely used failure criterion for anisotropic composite materials with different strengths for tension and compression) is expressed, in the Voigt notation [35], as follows:

*F_i_σ_i_* + *F_ij_σ_i_σ_j_* ≤ 1, (1)

where *i*,*j* = 1,2,…,6, *σ_i_* are the stresses, *F_i_* are the material strength parameters calculated directly from the stress limits (Table 6), and *F_ij_* are coupling coefficients (it was assumed that all are equal to −1) [36]. The ISF was calculated as the maximum value of the described three criteria.

**Figure 10:**
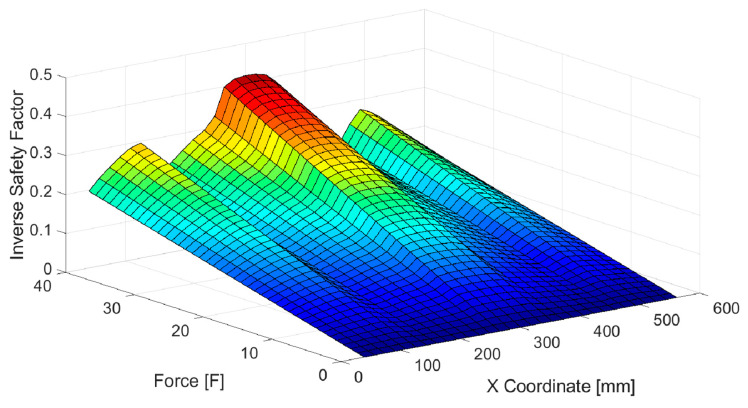
Surface plot of the inverse safety factor as function of the value and location of the acting force.

A surface plot of the ISF as function of the value and location (X-coordinate, see Figure 5) of the applied load is presented in Figure 10. As depicted in this Figure, the ISF is lower than 1, meaning no failure of the composite occurs for considered load values. Obviously, this factor increases with the increment upon the load. Also, as expected for the applied load cases, it is more likely for failure to happen when load is applied at the center of the panel and the risk is reduced when forces act over the stiffeners.

**Figure 11:**
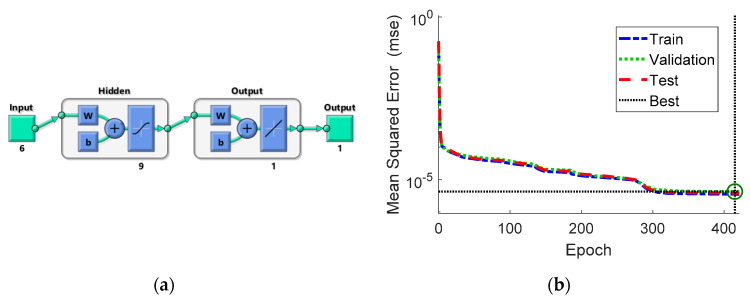
Trained artificial neural network (ANN): (**a**) architecture and (**b**) training performance.

An artificial neural network with two layers has been trained (see File S1). The ANN has got six inputs (strain values from the strain gauges, SG1–SG6) and one output (ISF). Sigmoid symmetric activation function is implemented in the hidden layer. The single neuron in the output layer has got linear activation function. As the idea is to perform on-line OLM, possibly using embedded devices, the goal was to find ANN with a simple structure (with as little neurons) as possible. The number of hidden neurons has been chosen by trial and error, while training and testing different networks with the number of hidden neurons between 3 and 20. ANN with nine hidden neurons has been chosen, diagram of which is presented in Figure 11a. Seventy percent of reference data were used for training, 15% for validation, and 15% for testing. The Levenberg–Marquardt algorithm [37,38], with random initial solution (normalized using the method of Nguyen and Widrow [39]), was used to train the ANN, the plot of training record error values against the number of training epochs is presented in Figure 11b. The best validation performance of 4.2275 × 10^−6^ was achieved at epoch 415.

### 3.1. Off-Line Testing of the ANN with Experimental Data

**Figure 12:**
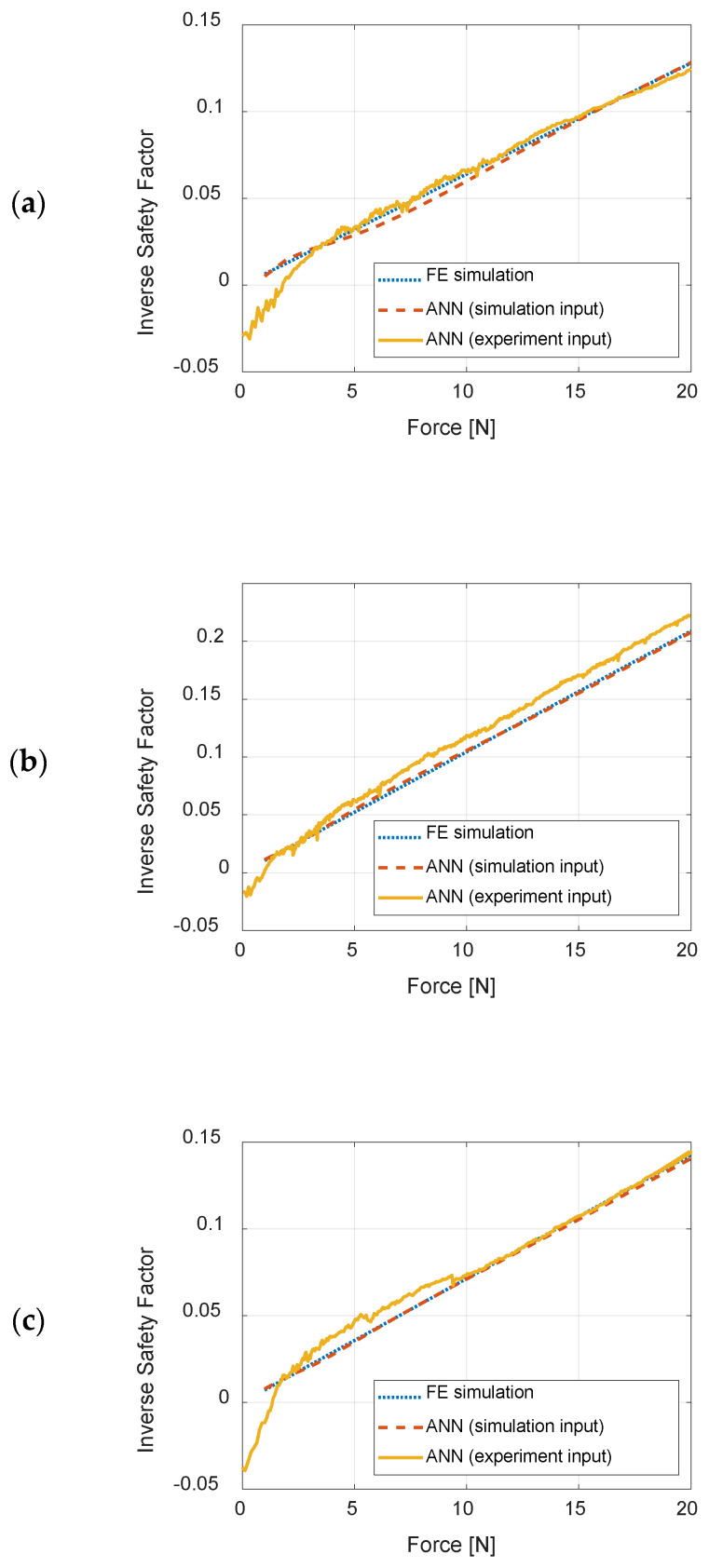
Results of testing ANN with experimental and simulation data: (**a**) load case LC1; (**b**) load case LC2; and (**c**) load case LC3.

The ANN was tested with the strain values obtained from the experimental measurements, the results being presented in Figure 12 for different load cases, LC 1, LC2, and LC3. The ISF estimated by ANN from experimental data was compared to ANN estimation with strain inputs from FE simulation, and to the exact value of ISF obtained from FE simulations. Root-mean-square errors between ISF estimations by ANN from strain data obtained from FE simulation and exact values of ISF (from FE simulations), as well as between ISF estimations by ANN from strain data from experiments and exact values, for each load case, are listed in Table 7.

As depicted in Figure 12 and Table 7, the ANN returns very accurate results, when its inputs are values obtained from numerical simulations, similar to data on which the ANN was taught, the root-mean-square-error does not exceed 0.0028. When strain values measured experimentally are given to the ANN as inputs, slight noise is visible however, the accuracy of the results can be considered as satisfying, as the root-mean-square-error is not greater than 0.0109. Noticeable differences can be observed for lower values of ISF, around 0, when the acting forces are of values less than 3 N. The probable cause of this situation is that the lower values are measured by the strain gauges, the greater the relative error of the measurements is.

### 3.2. On-Line Testing of the ANN

**Figure 13:**
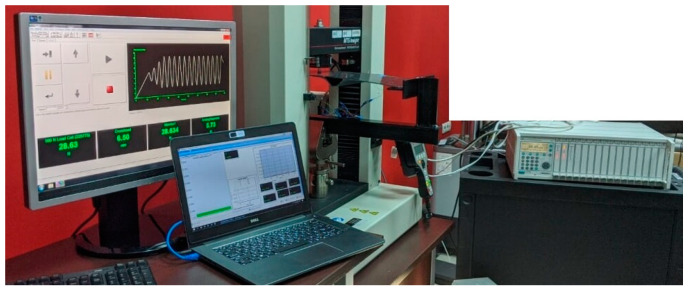
Experimental real-time testing of the ANN.

The ANN has also been tested in real time, where the inverse safety factor was computed by the data acquisition system based on the obtained strain values from strain gages during the experiment. A series of experiments were performed where load was applied at point LC2 (see Figure 6). Figure 13 shows one of the performed experiments (Video S1 and more detailed photographic documentation, Figures S1–S4, of the experiments are available in the Supplementary Materials).

**Figure 14:**
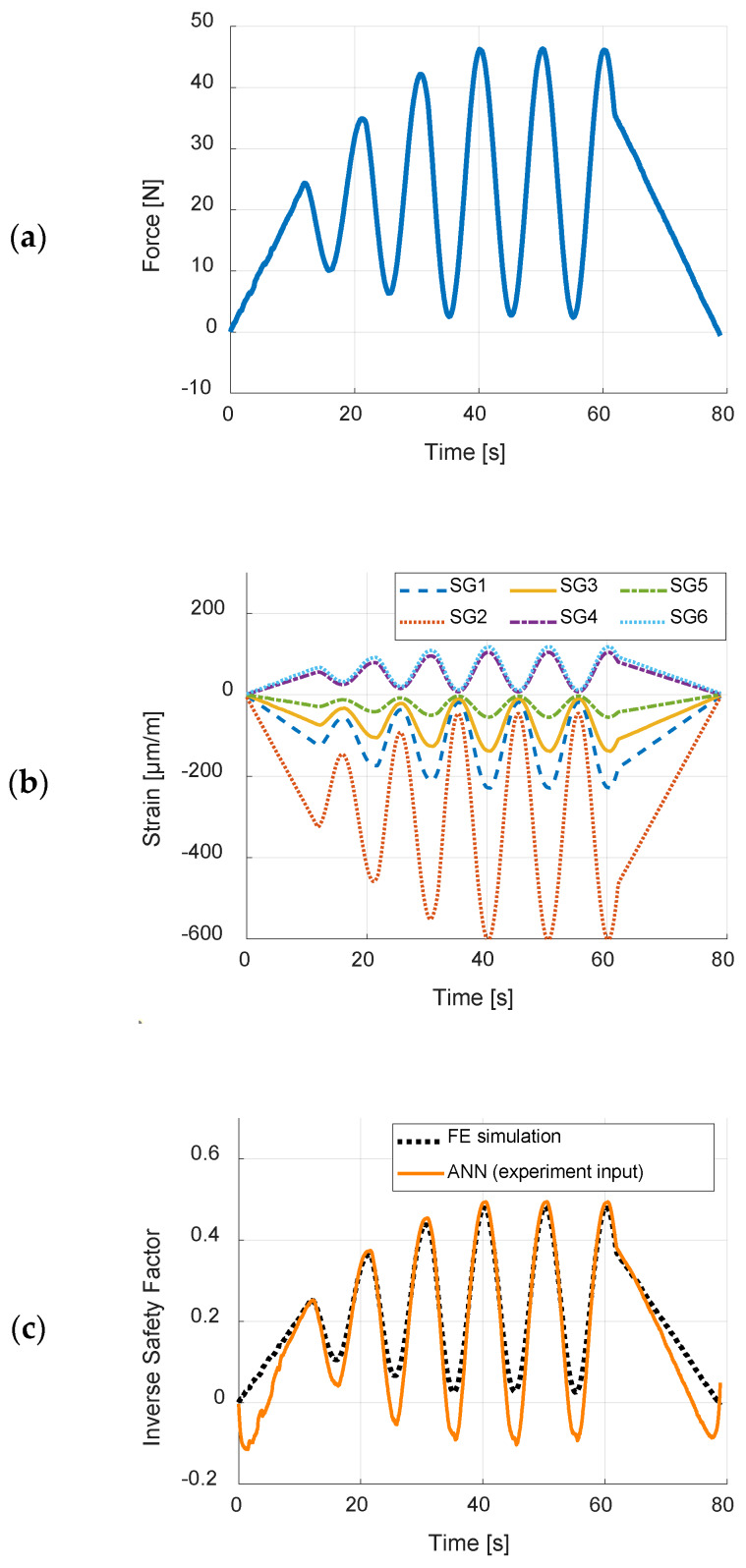
Results of on-line testing of the ANN: (**a**) time course of applied force; (**b**) time courses of acquired strain values from strain gages; and (**c**) comparison between time courses of ISF estimated from ANN and exact values from finite element (FE) simulations.

The experiment was driven by a crosshead displacement–time course. Time series of the force measured by the load cell and values acquired from strain gages (Spreadsheet S8) are presented in Figure 14a,b, respectively. Figure 14c presents the comparison of the ANN estimation of the inverse safety factor and its exact value (obtained from FE simulation, when inputting force values from Figure 14a), over time, the root-mean-square-error between those two plots is 0.0644. Similarly, as in off-line results, the ANN returns ISF values of quite good accuracy when the force values are significant, when low values of measured strain are inputted to the ANN, the differences between estimation and accurate results are noticeable.

## 4. Discussion

The presented results prove that ANNs can be employed in OLM processes, which are particularly important in aerospace industry, to reduce the number of sensors for strain measurements while maintaining the required accuracy.

The necessary condition to implement the method proposed by the authors is to have an exact numerical model of the structure to generate the training data for ANN. In the presented research concerning an omega-stiffened panel, detailed experimental verification of the numerical model was performed. Firstly, the computed material properties of the laminate (from properties of a single ply) were compared to experimental measurements of Young Modules of normalized samples (made in two different orientations), and an average relative error of 1.31% was achieved. Next, the accuracy of the whole finite element model was verified by a series of experiments in two steps. The first step, where bending stiffnesses in different points were computed, measured, and compared, gave average differences of 0.93%. In the second step, the strain distributions during different quasi-static loading, obtained from numerical simulations and experimentally from strain gages, were compared. The average root-mean-square-error from all measurements is of 3.19 µm/m, which is rather low, taking into account the maximum strain values. All the performed experiments showed that the verification of the numerical model can be considered successful. It has proven that both material and geometrical models of the panel are of high accuracy. In fact, the type and number of finite elements of the discrete model allowed to minimize the numerical errors and, hence having a high-fidelity model. The minor errors that were obtained are inevitable and might result from material or geometrical imperfections from the manufacturing process, as well as from the accuracy of the measuring equipment.

Although the strain–force relations are close to linear for each single load case, the main difficulty for the ANN was that it must work for many different load cases, without the knowledge where forces are applied. This complication makes the input-output relation of the ANN highly nonlinear, as indicated by Figure 10. In the presented example, the ANN method should result in highest ISF value for the whole panel, based only on six strain measurements. An ANN with only nine neurons in the hidden layer was trained. Both off-line and on-line testing of the ANN with experimental data showed that the obtained results are quite accurate, compared to numerical simulation results (considered here as exact). The biggest differences were obtained for very low (less than 0.1) values of ISF, where the ANN sometimes resulted in negative numbers that have no physical meaning. However, it can be assumed, that the very low values of the ISF are of low importance, and a simple conditional statement that if the ANN results with a negative number, the result should be taken as zero, would be sufficient.

Finding the right architecture of an ANN is not a trivial task. In the presented application the number of the neurons should be as small as possible, as the computations might by run on an embedded device (with significantly less computing power than a standard computer) when executing the on-line OLM process. However, too small number of hidden neurons will make the ANN a poor-quality estimator. In the presented case, training an ANN with a higher number of neurons might result in higher accuracy, however, too large number of hidden neurons often also results in decrease of results quality as the output fluctuates in the area between training points [40,41].

The number of sensor inputs to the ANN is also expected to be of crucial significance. In the presented case, six sensors, considered as relatively few, located in half-length of different surfaces of the model, seem to be sufficient, as the quality of the ANN output is satisfying. An analysis of influence of particular sensors on the predicted unmeasured data was out of scope of this work. The number of required sensors depends on the complexity of the structure, its shape and load conditions. There is no straight answer on how many sensors and where they should be placed on the structure for OLM using ANN, but strain distributions obtained from finite element model for different loading cases, engineering practice and judgement, as well as from trial and error approaches are helpful.

The on-line experimental testing of the ANN proved that the OLM using the proposed method can be successfully executed also in real time, with time varying strain data.

## 5. Conclusions

This paper presents an idea of implementing artificial neural networks to the process of operational load monitoring (of a complex composite panel—curved omega-stiffened composite panel), widely used in aerospace industry to monitor aircraft structures. Implementing ANNs allows to reduce the number of necessary sensors, whilst maintaining a high accuracy of the results. This could be particularly important if the structure is subjected to many different load cases during its normal service, and the number of critical points that should be monitored without an artificial intelligence approach is high.

The presented results validated the idea, moreover they proved that all the computations with ANNs can be successfully performed also in real time, if required. The research was focused on operational load monitoring, where current inverse safety factor of the structure was obtained over time during loading. Training the ANN to return other quantities, such as the maximal von Mises stress, location, and value of the acting forces, or displacement at a given point of the structure, would also be possible.

The developed algorithms and obtained results are considered by the authors as the starting point for future research that involves: (a) transferring the obtained knowledge to future applications of similar structures, (b) implementing multiscale modelling to the finite element model of the structure, (c) training ANNs with data from more complex simulations (e.g., fatigue damage and crack growth) and estimating the remaining useful life from strain data, (d) refining/updating the reference data obtained from simulation with experimental results, and (e) testing other machine/deep learning methods for OLM.

## Figures and Tables

**Table 1:** Ply orientation in different parts of the laminate (assuming even-symmetry).

Ply Number	Base	Reinforcement
1	0°/90°	0°/90°
2	−45°/45°	0°/90°
3	0°/90°	0°/90°
4	−45°/45°	0°/90°
5	0°/90°	0°/90°

**Table 2:** Orthotropic mechanical properties of a single ply and laminates.

Property	Single Ply/Reinforcement	Base	Base Material Rotated 45°
*E_x_* [MPa]	64,700	50,600	41,200
*E_y_* [MPa]	64,700	50,600	41,200
*E_z_* [MPa]	7171	7171	7171
*ν_xy_* []	0.040	0.249	0.388
*ν_yz_* []	0.340	0.266	0.217
*ν_xz_* []	0.340	0.266	0.217
*G_xy_* [MPa]	4000	14,800	20,300
*G_yz_* [MPa]	2662	2662	2662
*G_xz_* [MPa]	2662	2662	2662

**Table 3:** Experimental verification of mechanical properties of the composite rectangular samples.

Sample Number	*E_x_* (MPa) (0°/90°)	Relative Error (%)	*E_x_* (MPa) (−45°/45°)	Relative Error (%)
1	52,691	4.13	39,089	5.13
2	51,587	1.95	42,656	3.54
3	51,859	2.48	41,739	1.31
4	50,537	0.13	39,616	3.84
5	49,635	1.91	40,207	2.41
Average	51,261	1.31	40,662	1.31

**Table 4:** Comparison of numerical and experimental stiffnesses of the composite panel.

Load Case	*k*_num_ (N/mm)	*k*_exp_ (N/mm)	Relative Error (%)
LC1	6.48	6.47	0.2
LC2	4.27	4.32	1.2
LC3	5.87	5.79	1.4

**Table 5:** Root-mean-square-errors between numerical and experimental strain values (µm/m).

Strain Gage	Load Case LC1	Load Case LC2	Load Case LC3
SG1	14.83	2.54	3.49
SG2	5.69	3.69	6.95
SG3	1.54	1.55	2.44
SG4	1.57	2.02	1.25
SG5	1.27	0.60	1.41
SG6	1.56	1.44	3.55

**Table 6:** Orthotropic stress and strain limits for inverse safety factor (ISF) computation.

Direction	Stress Limit (MPa)	Strain Limit (µm/m)
Tensile *X*	200	3150
Tensile *Y*	200	3150
Tensile *Z*	12.5	200
Compression *X*	−125	2550
Compression *Y*	−125	2550
Compression *Z*	−42.5	3000
Shear *XY*	30	5500
Shear *YZ*	16	4800
Shear *XZ*	16	4800

**Table 7:** Root-mean-square-errors between exact and approximated by ANN values of ISF during off-line testing of the ANN.

Load Case	Simulation Data Input to ANN	Experimental Data Input to ANN
LC1	0.0028	0.0032
LC2	0.0016	0.0109
LC3	0.0013	0.0059

## Supplementary Materials

The following are available online at https://www.mdpi.com/1424-8220/20/9/2534/s1, Spreadsheet S1: experimental stiffness measurement at point LC1, Spreadsheet S2: experimental stiffness measurement at point LC2, Spreadsheet S3: experimental stiffness measurement at point LC3, Spreadsheet S4: experimental strain measurement at point LC1, Spreadsheet S5: experimental strain measurement at point LC2, Spreadsheet S6: experimental strain measurement at point LC3, Spreadsheet S7: ANN training data, Spreadsheet S8: measurement data and computed inverse safety factor from on-line testing of the ANN, File S1: MATLAB Code of the trained ANN, Video S1: experimental on-line testing of the ANN, Figure S1: photograph of panel mounted in the testing machine, Figure S2: photograph of acquisition hardware HBM during experiment, Figure S3: photograph of software during on-line testing of the ANN, Figure S4: screenshot of Catman Easy software during on-line testing of the ANN.
