# Supplementary figures and images for: Operational Load Monitoring of a Composite Panel Using Artificial Neural Networks
Source: Sensors (Basel). 2020 Apr 29;20(9):2534. doi: 10.3390/s20092534 (PMC7273206; doi:10.3390/s20092534)

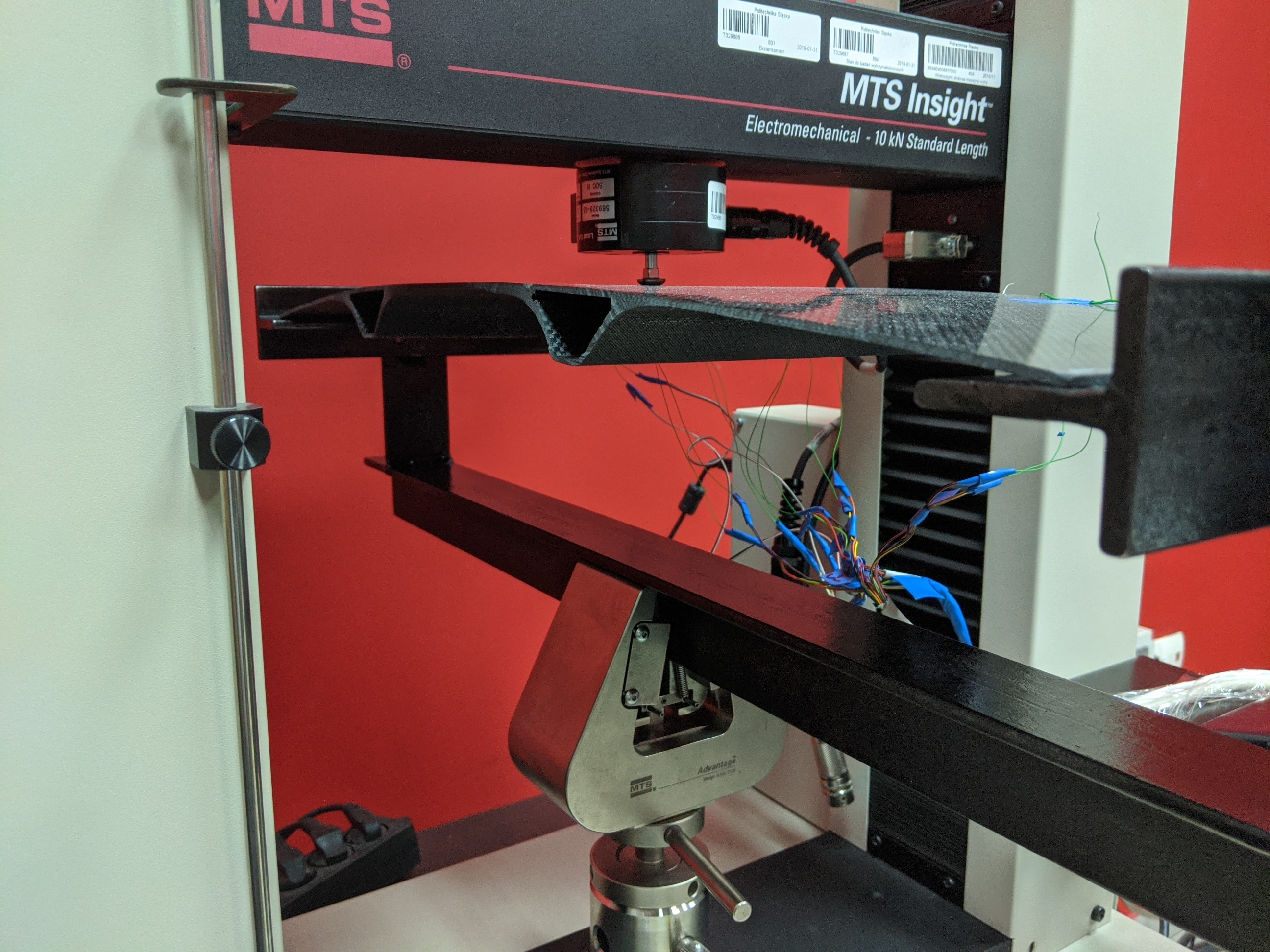

Supplement: Supplementary file 1 [file sensors-20-02534-s001.zip › supplementary/Figure1_panel.jpg]

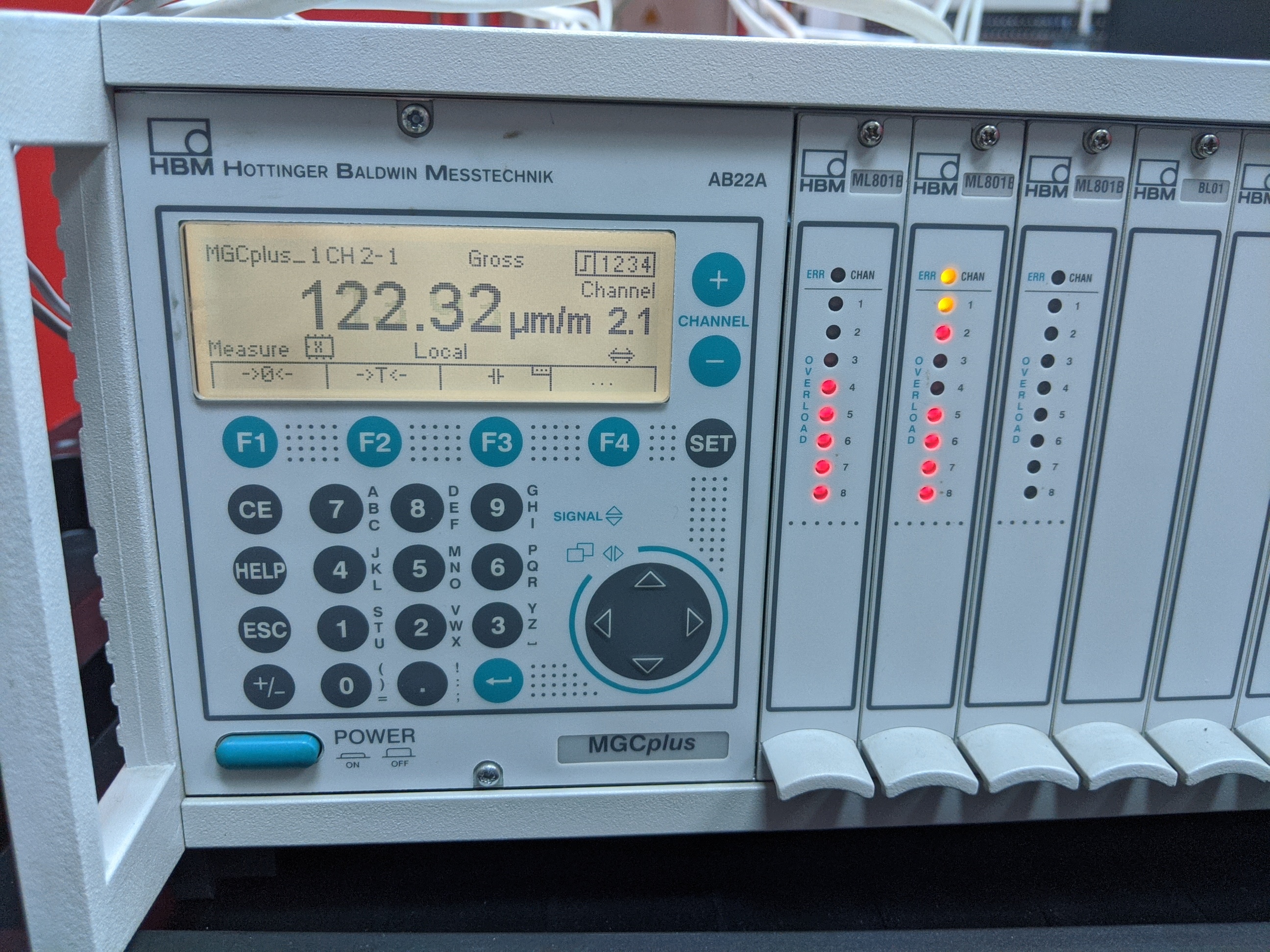

Supplement: Supplementary file 1 [file sensors-20-02534-s001.zip › supplementary/Figure2_HBM.jpg]

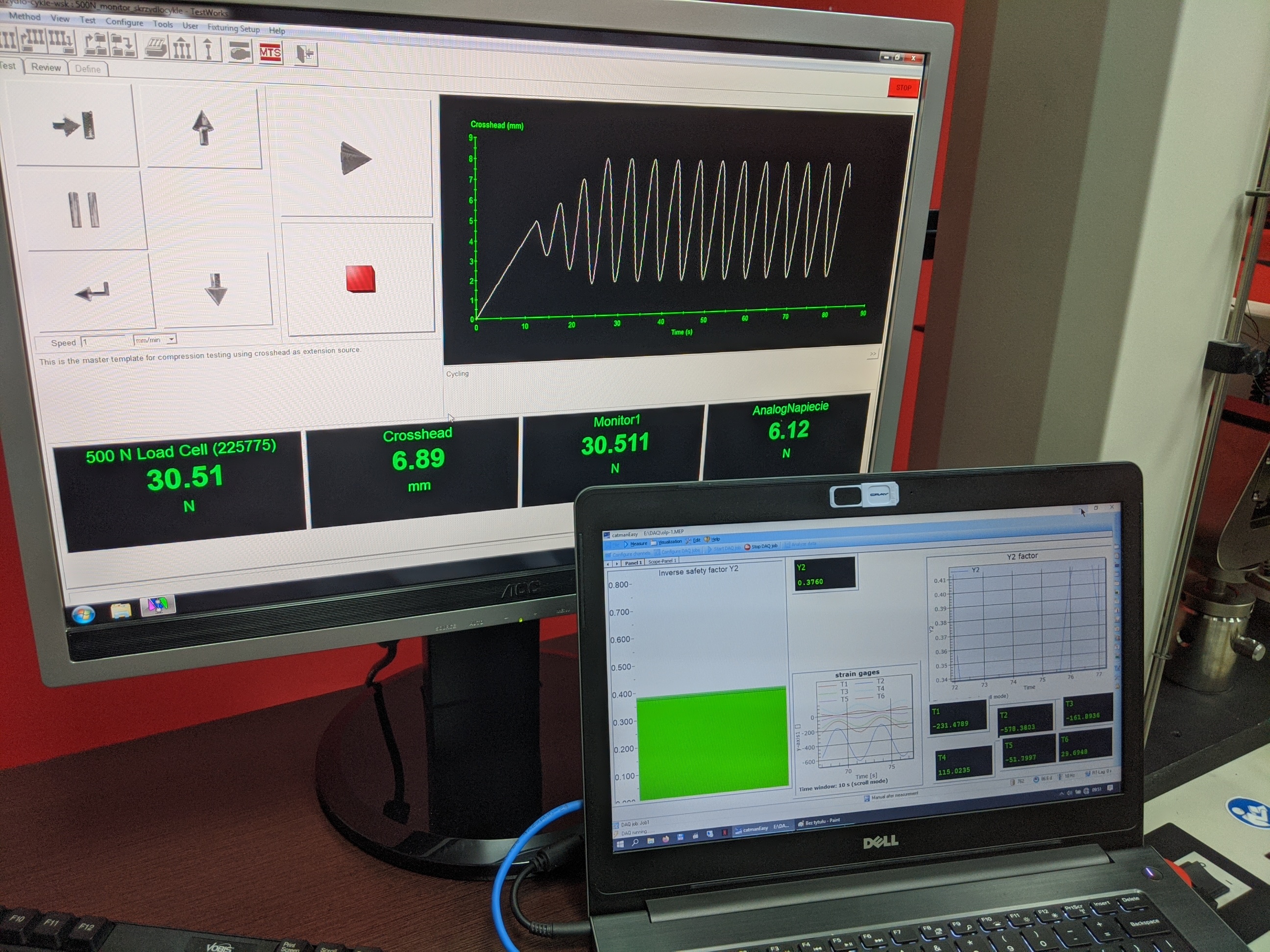

Supplement: Supplementary file 1 [file sensors-20-02534-s001.zip › supplementary/Figure3_software.jpg]

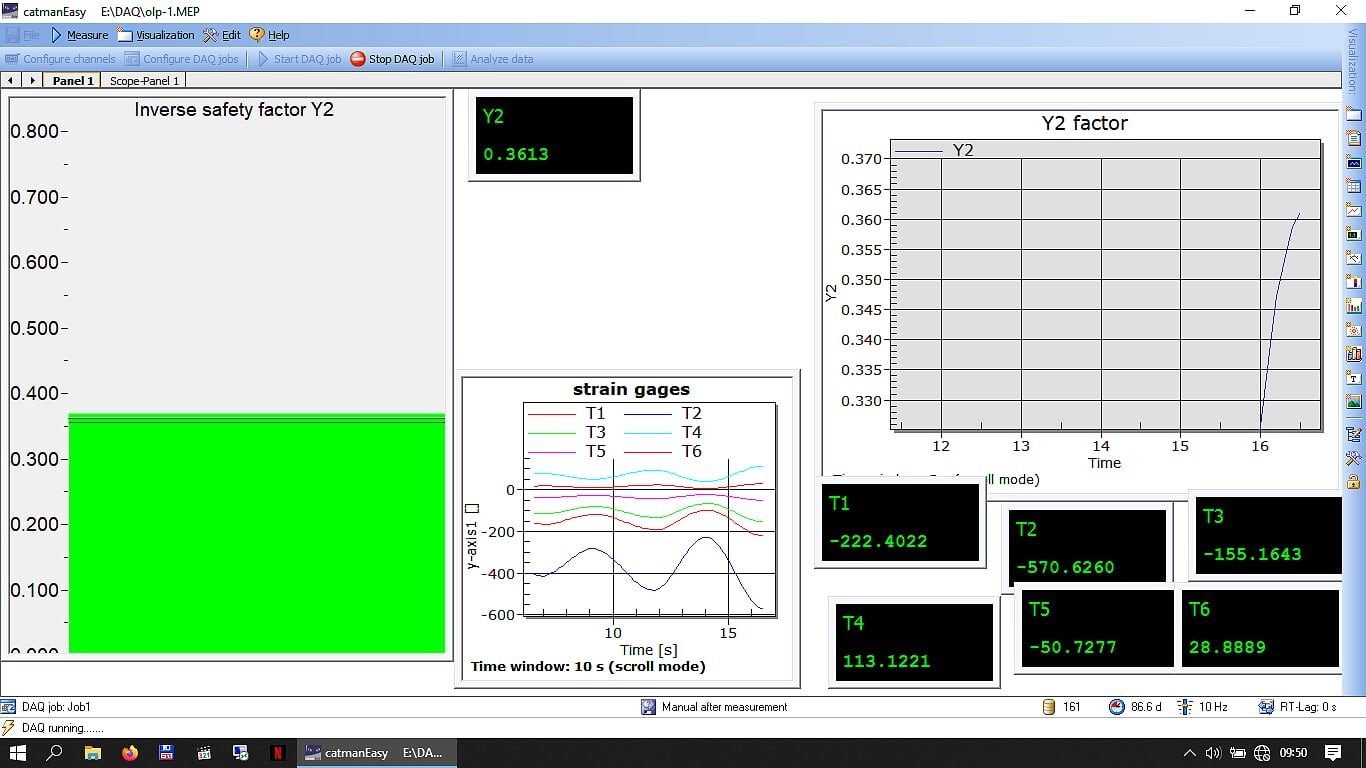

Supplement: Supplementary file 1 [file sensors-20-02534-s001.zip › supplementary/Figure4_catman.png]
